# A pilot qualitative study of New Zealand policymakers' knowledge of, and attitudes to, the tobacco industry

**DOI:** 10.1186/1743-8462-4-17

**Published:** 2007-07-25

**Authors:** Sheena Hudson, George Thomson, Nick Wilson

**Affiliations:** 1 Department of Public Health, University of Otago, Wellington Box 7343 Wellington South, New Zealand

## Abstract

**Background:**

The actions of policymakers are critical to advancing tobacco control. To evaluate the feasibility of using anonymous in-depth interviews to ascertain policymakers' knowledge about, and attitudes to, the tobacco industry, we undertook a pilot study involving New Zealand policymakers.

**Methods:**

Five politicians (from different political parties) and five senior officials, who were involved in tobacco control policy, were recruited for semi-structured, anonymous, face-to-face interviews.

**Results:**

Recruitment of appropriate senior policymakers was found to be possible. Interviewees were willing to answer questions fully and frankly about their knowledge and views of the tobacco industry.

The preliminary data from this pilot suggest that some New Zealand politicians appeared to see contact with the industry as similar to contact with other groups, whereas the officials indicated at least a different style of relationship. Only one politician knew if their party accepted funding from tobacco companies. All but one of the interviewees thought that promotion of tobacco to under-16 s still occurs, albeit indirectly. The interviewees' knowledge of the investment in tobacco industries by New Zealand government agencies was low or absent.

While most of those interviewed showed scepticism about tobacco company public relations efforts, this was absent in some cases. There was a wide understanding that the tobacco industry will use many tactics in the pursuit of profit, and to counteract government efforts to reduce the harm from smoking.

**Conclusion:**

In-depth anonymous interviews appear to be feasible and can be productive for exploring sensitive tobacco-related policy issues with policymakers. The preliminary data from this group of New Zealand policymakers suggest important knowledge gaps, but also general distrust of this industry. From a tobacco control perspective, the results may suggest a greater focus by advocates on the funding of political parties by the tobacco industry, and on government agency investment in the tobacco industry.

## Background

Advocates for public health need to know about the context for relevant policies [1-5]. To enable researchers and health advocates to inform tobacco control policy, research must increase the understanding about the opponents and allies of effective policies [6]. For tobacco control, this includes information on the way policymakers regard the tobacco industry. In New Zealand this industry appears to be a major barrier in the advancement of tobacco control [7].

The published research on policymakers' knowledge of, and attitudes to, the tobacco industry is largely limited to North American, and to quantitative surveys with fully structured interviews [8-12]. Little qualitative research has been published on policymaker knowledge about the *tobacco industry*. Qualitative studies of the wider tobacco control policy process, using in-depth interviews, has been largely limited to the state and local level in the USA and Australia, except for one study at the national level in the USA [13]. Except for one study each in Australia [14], and the USA [15], such studies have been limited to legislators rather than others, such as officials [16-23]. In addition, case study approaches (eg, [24,25]) have provided valuable context for policymakers' knowledge of, and attitudes to, the tobacco industry.

The aim of this study was to evaluate the use of in-depth interviews with a small group of New Zealand policymakers, on their knowledge about, and attitudes to, the tobacco industry. In particular, we were interested in evaluating this approach with politicians and senior officials involved in, or with some possible involvement in, tobacco control policy and legislation.

## Methods

### Selection and recruitment process

This pilot study aimed at interviewing ten participants. Potential participants were selected from (i) current politicians who currently had, or had previously had some role in tobacco control policy in the past ten years, or had commented publicly on such policy in that period; and (ii) current senior government officials who were in a position to affect or comment on tobacco control policy.

A search in the New Zealand print media (using the database Factiva.com) and the parliamentary record for politicians' comments on tobacco control since 1996, identified 17 politicians. The further inclusion of those who fitted the above criteria resulted in a total list of 24 politicians. The criteria for the creation of a list of officials for potential interviews included seniority, closeness to the tobacco policy process, and potential ability to affect policy. An initial group of seven officials meeting the criteria were identified.

From these 31 names, a priority list was established, on the basis of having an even representation between politicians and officials, representation by gender and ethnicity, and from a range of political parties. A further selection was made to ensure a balance, where possible, between politicians whose comments indicated positive or negative attitudes to the tobacco industry.

Permission was obtained from the University of Otago research ethics system for the conduct of the anonymous interviews. The recruitment process included: (i) An initial approach by letter, followed up by a phone call within a few days; (ii) Clearly advising potential interviewees of the anonymity and confidentiality of the interviews and study results, in the letter and verbally; (iii) Giving potential interviewees complete availability of times for interviews, including before and after 'normal' office hours; (iv) Being politely persistent in the recruitment process (and humorous where appropriate) and; (v) Offering a short interview time where necessary.

### Interview schedule and process

A semi-structured interview schedule was prepared to address the research aims, derived from the literature and previous research knowledge. The questions were open in design and allowed some flexibility for interview time constraints. They allowed the respondent to expand on any particular or more relevant points of interest. The questions were kept unaltered during the interview series. Consistent interview prompts were used, where needed.

The interviews took place at the participants' work place, between May and August 2006. The time taken was between 25 and 45 minutes. Interviewees were reminded of the study details (including the interviewee anonymity and ability to stop recording the interview) and a short period was spent building some rapport and trust.

All interviews were audio recorded, and were transcribed by the interviewer (SH). Beyond the stated anonymity and confidentiality of the interviews and study results, the fundamental approach of the interviews was to protect the interviewees' rights [26]. The relationship between the researcher and the interviewee in the interview was seen as important, and the interviewee was seen not a distant aseptic 'research object', but as a real person whom the interviewer recognised and treated as such. As part of the approach, any opinions or bias of the researcher were either withheld, or made clear where appropriate or necessary. This approach puts into effect the codes of the New Zealand Association of Counsellors and National Oral History Association of New Zealand, which were taken as guides for the work [27,28].

### Data analysis

The data analysis methods, and some of the possible themes or codes, were determined by our choice of a semi-structured interview format. This format meant that we used template analysis to identify themes [29]. Template analysis lies between content analysis (where there is a predetermined list of codes) and grounded theory (where there are no predetermined codes). Memos made after each interview gave a preliminary identification of themes. These themes were adapted and changed, as further themes emerged during the transcription and coding process. The material was coded using the NVivo software programme. The coding results were checked against the audio recordings and transcripts by second researcher. We included as wide a range as possible of verbatim transcripts in the article, to allow the reader to evaluate the material for themselves.

## Results

### Recruitment and interview feasibility

Five out of the first six officials who were approached agreed to be interviewed. The one who declined was on extended leave. Of the first six politicians approached, three declined immediately, and one declined after three failed attempts to keep an appointment, leaving two interviewees at that stage.

A further group of six politicians were approached and three agreed to participate (giving an overall response rate for politicians of 5/12 or 42%). For the others, one politician's secretary thought the inquiry should go no further, and two declined and recommended other members of their caucuses whom they thought more knowledgeable about the tobacco industry. The two who were recommended were also on the list, and had already agreed to take part.

The ten participants consisted of politicians from five different political parties (out of the eight represented in the 2006 Parliament), and managers or senior advisors from the three Ministries of Health, Education and Social Development. Persistence was needed to ensure access to interview the politicians. It was necessary to be flexible with their appointment times, as they could be called urgently to government business, or be late because of extended caucus meetings.

Interviewees were willing to answer questions fully and frankly about their knowledge and views of the tobacco industry. The time and effort spent on establishing rapport and trust appeared to be successful in obtaining frank and open answers and comments. Participants often showed genuine surprise or shock, and were willing to make statements that might not be in keeping with their political party or organisational policy. As far as the interviewer could judge, and as could be heard from the audio recording, any reticence appeared to be due to lack of knowledge about the topic in question.

### Contact with the industry

Participants were asked if they had had any contact with tobacco industry personnel. For four of the participants (three politicians and one official), industry contact was regular, and they saw it as part of their job. One politician described their role as being the '*contact person for the ...party'*. A second official described the experience of being approached by a 'salesperson' from the tobacco industry, who wanted the official's support for a community project being funded by his tobacco company. Five other participants had had one significant contact, but it was not on-going, and one official stated he never had contact.

Politicians and officials appeared to view contact with the industry in different ways. On being asked whether contact with tobacco industry personnel would be any different from that with other agencies or industries, the politicians in the study stated that their contact would be no different. In contrast, most of the officials were very clear that their contact would be different, for reasons such as:

*'I view them as producers of a hazardous substance, so I would have different mind set*.'

'Because most of the other agencies I deal with are either government agencies or non-government organisations, charitable organisations and so on. The similar organisation [to the tobacco industry] would be with the food industry and I do have contact with the food industry. But I think the tobacco industry is slightly different to the food industry, and I think my relationships would be quite different.'

### Knowledge of the tobacco industry and its associations

The interviewees' knowledge of the investment in tobacco industries by New Zealand government agencies [30] was low or absent. All except three did not know of, and were surprised to hear of, the investment. Of the three, one had no firm knowledge of details, and two only showed any knowledge after denials and prompts.

When the politicians were asked about their party's policy on accepting money from tobacco companies, only one said that their party had a formal policy to not accept money from tobacco companies. According to three politicians from other parties, those parties appeared to have informal policies that such funding had not and would not happen. The remaining politician said he thought his party would not accept money from tobacco companies, but would now check on this.

The three participants closest to the tobacco policy process (based on their statement of tobacco policymaking being an important part of their job) agreed that they needed to have up-to-date knowledge about the actions and nature of the tobacco industry, when making policy decisions. This included:

'Evidence from overseas on the way that they operate ...and all the efforts around the world to legislate the tobacco industry.'

### Attitudes to public relations initiatives by tobacco companies

For most (but not all) interviewees, the general subject of tobacco company public relations activity provoked stronger reactions than any other area of questioning. Seven of the participants were more forceful in addressing this subject than when answering any other question. They were sceptical of the motives of the tobacco companies in any activity relating to health outcomes (such as the tobacco company support of the Life Education Trust in New Zealand, an organisation that seeks to provide drug education in schools). For instance:

'No business is going to invest its money in activities that go against its own interests.'

'They wouldn't be doing it if there was not some spin-off for their product ... I find it remarkable that the industry can come out with statements like being "socially responsible." '

'I think there are conflicting messages...it is good that they are doing those things, but you would need to follow the logic of that through ...one can't on the one hand promote a health message and on the other hand market a product which is responsible a considerable amount of ill health in the community.'

'They are putting on some nice clothes, so they can't be seen for what they are.'

However, one interviewee suggested that a tobacco company donation to a nominally health-focused charity was:

'given in good faith ... When I spoke to people I had contact with in the tobacco industry about that, they were very clear in their minds that they were doing good work and that that was a positive.'

### Attitudes on associations with the tobacco industry

Interviewees were asked what risks there might be to the government, when it and or its agencies were associated in any way with the tobacco industry. Seven of the participants, including three politicians, thought that if such contact extended to co-operation, it would be inconsistent with present government policy. They made clear statements on this, such as:

'I think that there is a risk of influence, in terms of the tobacco industry promoting their products, which is inconsistent with the smoke free policies, but also with the health of New Zealanders.'

'If you are talking about a healthy and safe environment for all New Zealanders, and then you are deliberately involved in practices that undermine that, then you are not putting your money where your mouth is ... you've got conflict going on.'

'Some companies you don't want to be in partnership with.'

### Knowledge and attitudes around tobacco company marketing

The interviewees showed little knowledge about the detail of tobacco marketing. Only one was able to volunteer any knowledge of tactics for marketing to women or particular ethnic groups. However, across all the interviews, their answers did suggest some underlying understanding of what might be happening in tobacco marketing. When asked whether they thought that tobacco companies in New Zealand promoted tobacco to children under 16 years of age, only one participant stated that they did *not *believe that there was any kind of promotion. The *sale *of tobacco products to those under the age of 18 is illegal, and one purpose of the *Smoke-free Environments Act *is:

*'to reduce the social approval of tobacco use, particularly among young people, by – (i) imposing controls on the marketing, advertising, or promotion of tobacco products and their association with other products and events*' [31].

Nine of the participants considered that there was tobacco promotion to under-16 s, even though this appeared to be against the general aims of New Zealand government policy. They also were aware of what they regarded of the subtleties of such promotion. The consistency and depth of their attitudes on this is suggested by such statements as:

*'I don't think they do go out and specifically target under-16 s, but ... I think it is actually very difficult to not ... cross the line'*.

'I think they do it through subtle marketing and peer pressure, and through availability... and I think little shops still market to children. Big shops, by which I mean supermarkets, I think, are less likely to display and market to children, although cigarettes are still on display.'

*'I think they don't do it as [much as] they certainly once did, but I'd be in no doubt that they'd be not too unhappy with any under-16 year old who starts smoking either*.

'I am sure there will be, deep down, perhaps a strategy on how they get their next generation of customers. But whether that's targeted to them or not, I don't know. There have certainly been incidents of products sold with cigarettes that would indicate that they are targeting younger people...and we've stopped that.'

*'Obviously there's subtle marketing efforts, and the industry are using them'*.

'*Talking to the people from BAT [British American Tobacco], I don't think they would consciously do that, but if you are going to [market] vigorously, obviously it can't escape people under 16*'.

In answering this question, four officials expanded on their attitudes on tobacco marketing, suggesting the power of video, television and movies to influence tobacco use by young people. The comments included:

'One thing that I feel strongly about [tobacco marketing]. That is the influence of movies. ... it sort of promotes the view that smoking is OK and acceptable.'

'There are still films made with people smoking as a social norm, and that has a significant influence.'

'Have we got tobacco role modelling going on particularly in television and the movies? And the answer is yes ...and does that influence youth behaviour? The answer is yes. Are tobacco companies tied up in product placement? I understand they are.'

### Attitudes on tobacco industry efforts to counter health protection activities

The participants were asked if they thought that tobacco companies deliberately set out to counteract government efforts to reduce the harm from smoking, through legal or official processes. All ten participants believed that the tobacco companies would use many different tactics to promote their own interests that were contrary to the health promotion and protection aims of the government. In answer to the question, comments from the politicians included:

'*Yes. Their mission is to sell tobacco*.'

'I am sure they look for other avenues, as do accountants when we change tax law, as we change regulations on any industry, they will always be those who wish to push the boundaries. Yes, I guess they have challenged government in terms of the process and as does any organisation. They will use the law and the legal process to promote their company objective, and that's increased profits and returns to shareholders.'

'I remember one of the South American doctors, employed by BAT, that came out and lectured all around the place and minimized the harm caused by tobacco... I think that they sprinkle a variety of medical professionals who have basically been captured ... paid off...absolutely.'

The officials in the group were also explicit in their attitudes on this:

'*They have been reasonably vigorous lobbyists against, for example, smokefree legislation. They have been fairly vigorous over time in terms of trying to combat medical evidence, with respect to say, the illness that is caused by smoking.'... 'I think they would certainly put pressure on groups who oppose them.'*

'It's just business isn't it ... it is surviving and in business you are trying to make a profit and trying to make the best money possible and you'll do what it takes to do that.'

'They use a variety of approaches ...both questioning research, doing their own researching [on] regulatory measures, challenging regulatory measures, challenging government processes.'

'At times they have been successful in challenging [the official] process, and that further delays [the process] and that means we've got to concentrate on very good process ...or we get challenged at any possible slip-up in process. And that [level of] challenge wouldn't normally occur in most other [health policy areas].'

### General attitudes to the tobacco industry

A more open question, asking interviewees more generally about their attitudes to tobacco companies, produced answers from all the interviewees. These contained both uncritical and critical aspects. For instance, one politician's comment appeared to assume that the industry was within the limits of 'normal' business, and that it showed some social responsibility.

'They are like everybody else. They have a job to do ... they sell tobacco. ... they do have that element of social responsibility there.'

Seven responses were wholly critical, and these ranged from mildly so, through to extremely forceful. Criticism came from both politicians and officials:

*'At one level I don't regard them as doing anything of great value, but then I regard the advertising industry in the same category'*.

'They are huge multinationals, and operate as such in an area of high profit, high taxation, high levels of regulation, but ultimately one that is still sanctioned by the vast majority of governments. [An industry] that we have become increasingly aware of, [which] produces harm for societies through direct and indirect utilization of the product, and... the only product that used as recommended will harm you.'

'I think they should all just shut up shop really ... [laughter]...and get into some other industry that's actually constructive and helpful ... I don't know how people can bear to work in them, I mean to be honest ... how could you?'

'Their product is harmful ... and whether they are good at being harmful or bad at being harmful ... they are harmful.'

'Their business is death. It is the sale of death, and for that reason I would like them out of the country and I believe that by so doing we will set an example that many other countries will follow.'

'Well, they are a scourge really ... they are the worst extreme example of global corporitisation.'

One interviewee felt that one word could fully describe tobacco companies:

*'Pariahs'*.

## Discussion

### Issues of methodology

While recruiting and interviewing senior policymakers was found to be feasible and productive, in our sample the officials were much easier to access and recruit compared to the politicians. There are a number of possible constraints on politicians being interviewed about this topic. These include greater 'gatekeeping' around them, compared to officials, less awareness or trust in the protection of anonymous interviews, and even less available time for interacting with researchers. Aides and personal assistants may believe that a potentially controversial subject could have negative consequences for the politician, and may block access to them.

Once past the 'guardians', recruitment may be hindered as the politicians may themselves be concerned with potential consequences about speaking out on such a subject, even in anonymous interviews. All national-level New Zealand politicians potentially have very large workloads, and it may be a temptation for them to put research in the 'able to be postponed or cancelled' tray.

The elements of an interview that makes them productive (including enabling interviewees to be open and frank) include the interview content and style, the interviewer, and the context of time and place. While we found that the anonymity of interviewee appeared to be an essential element for most of our sample, other essential elements may be less tangible.

The methods used by an interviewer to build trust, and the interviewer's necessary attributes, may vary from interview to interview. The attributes may include interviewer experience, confidence, and training, and the ability to listen, empathise and put at ease [32,33]. Being non-judgemental can be essential as part of making interviews feel 'safe' enough to speak out. In many cases, an interviewer may need to connect in some way with the interviewee's emotions, in order to get them to open out. Revealing some of themselves is often essential for interviewers. An enthusiasm for the subject matter of the interview helps. Such methods and attributes are in addition to the essentials for interviewers. Primarily, the ability keep the whole pattern of the interview in their minds while processing the interviewee's comments, and preparing further appropriate prompts or questions. In addition, sufficient charm, and sufficient mastery of the subject matter on the interview, are essentials for an in-depth interviewer.

The use of semi-structured in-depth interviews, rather than structured ones, allows fuller understanding by interviewees of questions, and much greater intricacy and subtlety of answers. Probing for further information by interviewers can be better adapted to the answers given. Such interviews give participants the opportunity to give answers with mixed opinions. The potential disadvantages of the longer time taken for analysis have been somewhat offset by the increased use of computer assisted qualitative analysis programmes.

### Attitudes to the tobacco industry

The politicians and officials had very different attitudes on their *contact *with the industry, with politicians appearing to see such contact as similar to contact with other groups, and officials indicating at least a different style of relationship. This difference may reflect a greater detachment by the politicians, or a perceived need by them to treat all parties in the same light.

However, there was some general similarity of attitudes, with all participants believing that the interests and activities of tobacco companies were contrary to the health objectives of the New Zealand government. All but one of the participants were clear in their attitude that promotion of tobacco to under-16 s still occurs, albeit indirectly. Their position is supported by the current exposure of young people in New Zealand to: (i) product displays in shops; (ii) tobacco advertising in imported magazines and on satellite television; (iii) and various forms of other marketing [34].

Against the general scepticism of tobacco company intentions, there were some different attitudes. Of note is that one interviewee did not consider that there was tobacco promotion to under-16 s, and three interviewees were not concerned about the level of contact between government and tobacco companies. One interviewee appeared to see aspects of social responsibility in the companies' behaviour, which contrasts with: research evidence [35-38], media portrayal [39], much of public opinion [40-42], and international official opinion [43,44].

### Limitations of the study

This study was a small pilot, with the main intention being to establish the feasibility and utility of the approach and qualitative methods used. As such its research findings are only indicative, and may have limited generalisability to the policymaking community in New Zealand.

A further limitation, arising from the use of a small sample of anonymous interviews, is that particular comments and their significance cannot be better described by being tied to particular parties or government agencies, or set in the context of the interviewee's ideology. Much larger studies would be needed before such analyses may be possible without endangering anonymity. More reliable results may also require repeat interviews over time that allow for a building up of trust with the interviewer, and work that links the policymakers' attitudes with their statements in the media and their voting behaviour on tobacco control issues.

### Suggestions for action by tobacco control advocates

These preliminary findings suggest that policymakers need to be better informed on the detail of tobacco industry activities, and that government investment activities need to be publicised. Publicity appears to be needed regarding policies on the funding of political parties by the tobacco industry, and the partys' relationships with the tobacco industry in general. The lack of clarity about political party policies indicates that tobacco control advocates may need to raise this issue with politicians.

The uncritical comments about tobacco companies, and lack of scepticism about their activities by some interviewees, suggest a need for more active provision to the public and policymakers of information about the nature of tobacco industry activity. Media campaigns on tobacco industry behaviour [45-49], and the accompanying media comment and coverage, may have the additional benefit of informing policymakers. It has been suggested that such 'mass media campaigns ... appear to be critical in preparing the ground for other measures aimed at fundamentally changing how the tobacco industry and its products are regulated' [40]. An official New Zealand tobacco industry denormalisation policy may have a number of benefits for health, including helping further change policymaker knowledge and attitudes [50].

### Suggestions for future research

A further larger qualitative study of policymakers' knowledge and views of the tobacco industry based on this pilot would give fuller and more generalisable results. Such a study of policymakers' knowledge and attitudes of the tobacco industry, with higher interviewee numbers, could also explore the relationship of the policymakers' ideology, training and sector background to their knowledge and attitudes [8,51].

In further studies, the gathering of documentary evidence would provide context for the interviewee comments, and could include government policy statements, official advice, party policies, and media coverage of the tobacco industry. An additional quantitative survey of officials could also lead towards more generalisable ideas on the influence of the tobacco industry-related knowledge and attitudes of policymakers, on their decisions about tobacco control policy.

## Conclusion

This pilot study found that, with appropriate methods, it was possible to recruit and successfully interview a diverse sample of New Zealand tobacco control policymakers about the tobacco industry. The interviews indicated strong attitudes about the activities of the industry. If these are widespread, advocates could widen and build on this emotional force. In particular, tobacco control advocacy work appears to be needed to better inform policymakers about the implications of government investment in the tobacco industry and the funding of political parties by the tobacco industry.

## Competing interests

Two of the authors (GT, NW) have undertaken contract work for a range of organisations involved in tobacco control including: ASH NZ, NZ Cancer Society, NZ Smokefree Coalition, and the Ministry of Health.

## Authors' contributions

GT conceived the project and developed it with SH. SH did the recruitment and interviewing. SH and GT analysed the data, and all authors were involved in writing the text. All authors read and approved the final manuscript.
